# Supplementary material for: The integrated stress response is tumorigenic and constitutes a therapeutic liability in KRAS-driven lung cancer
Source: Nat Commun. 2021 Jul 30;12:4651. doi: 10.1038/s41467-021-24661-0 (PMC8324901; doi:10.1038/s41467-021-24661-0)
Supplement: Supplementary file 1 — Supplementary Information [file 41467_2021_24661_MOESM1_ESM.pdf]

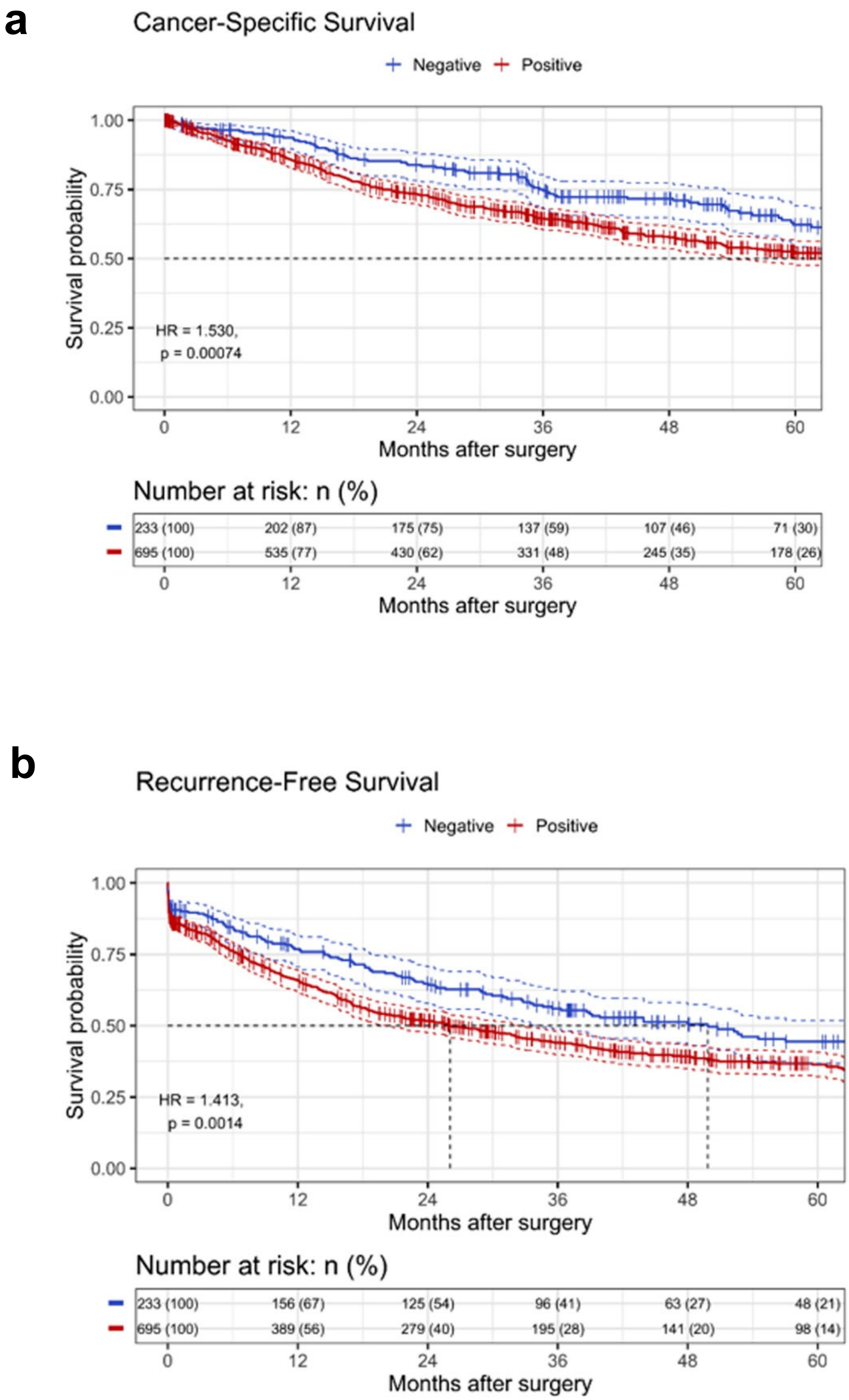

Supplementary Figure 1. p-eIF2 prognosticates poorer survival of 928 patients with primary LUAD after surgery. Kaplan-Meier survival curves of p-eIF2 expression for patients' Cancer-Specific Survival (**a**) and Recurrence-Free Survival (**b**). Statistical significance was determined using log-rank test (two-sided). Confidence intervals are represented by the dashed lines around the survival curves, in the representative colour. HR=hazard ratio.

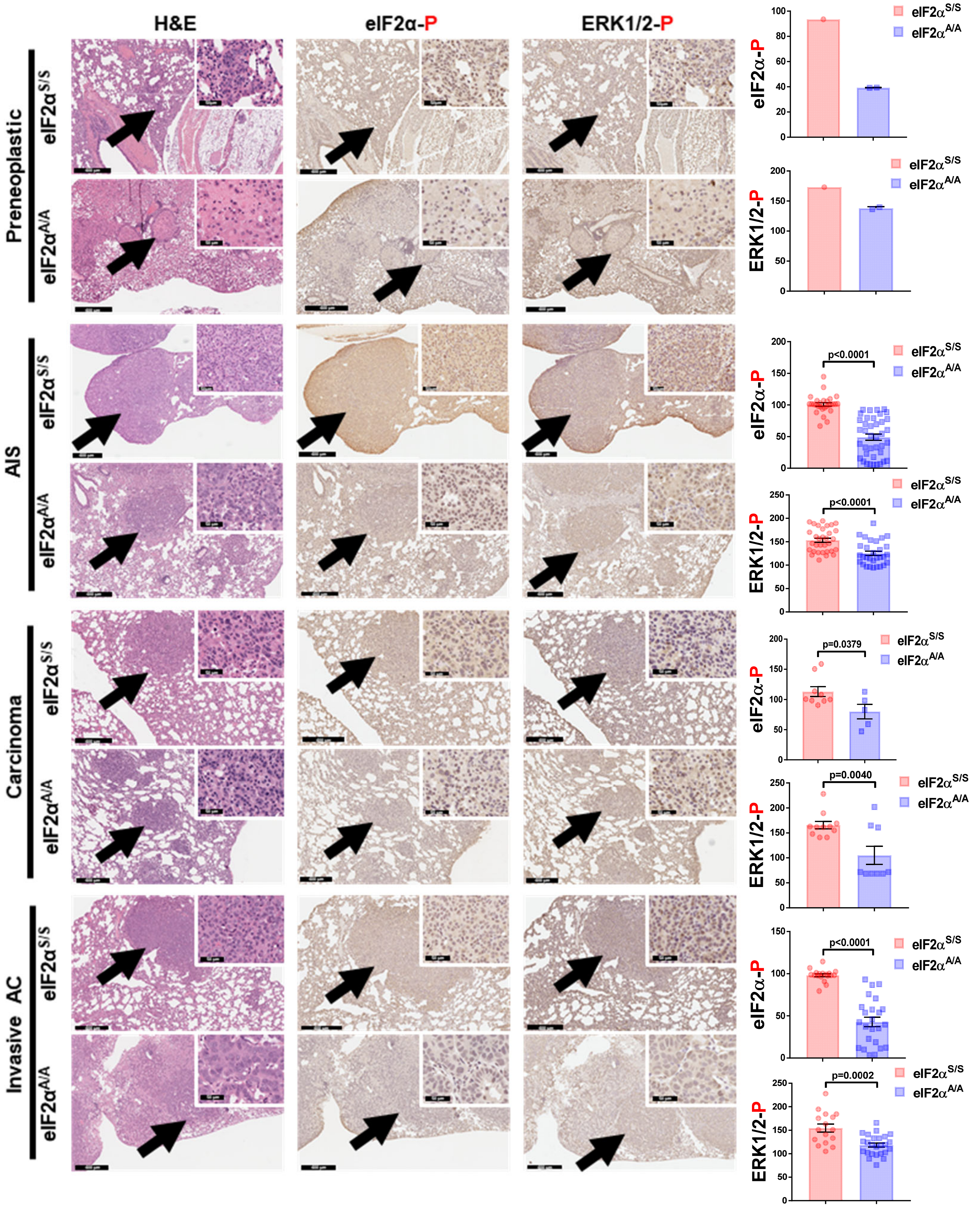

Supplementary Figure 2. p-eIF2 ablation decreases p-ERK regardless of histological type of mouse KRAS G12D tumors. H&E staining and IHC analyses for p-eIF2 (eIF2α-P) and p-ERK (ERK-P) in mouse KRAS G12D eIF2α$^{S/S}$ (n=5) and eIF2α$^{A/A}$ (n=5) lung sections with tumors of different histology. The graphs indicate the H-scores of p-eIF2 (eIF2α-P) and p-ERK (ERK-P) for each type of lung tumor at 20 weeks after KRAS G12D induction in eIF2α$^{S/S}$ and eIF2α$^{A/A}$ mice. Scale bars correspond to 400 μm and 60 μm of core and enlarged tumor images, respectively. Statistical significance was determined using two-tailed unpaired *t*-test. Data represent mean ± SEM.

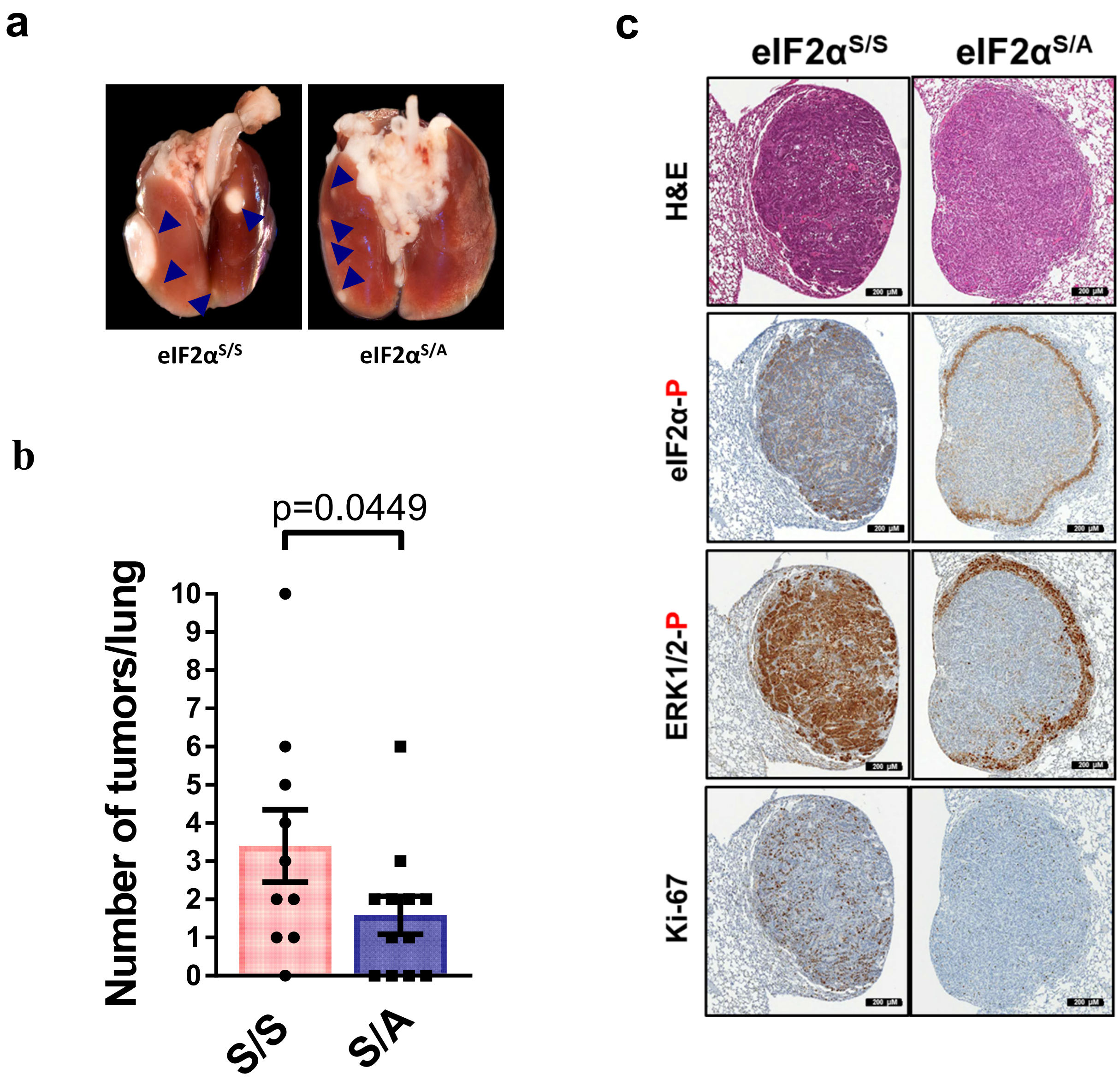

Supplementary Figure 3. p-eIF2 promotes urethane-induced lung carcinogenesis. (a) Representative images of KRAS lung tumors in WT eIF2α mice (eIF2α$^{S/S}$) as well as mice with a heterozygous germline S51A mutation of *eIF2S51* (eIF2α$^{S/A}$) after 40 weeks of urethane treatment. **(b)** Number of macroscopic tumors formed in mouse eIF2α$^{S/S}$ (n=10) and eIF2α$^{S/A}$ (n=12) lungs. Data represent mean ± SEM. Statistical significance was determined using one-tailed unpaired *t*-test with p= 0.0449. **(c)** Reduced p-eIF2 (eIF2α-P) in urethane-treated mouse lung tumors correlates with decreased p-ERK (ERK-P) and decreased proliferation (Ki-67). Scale bars correspond to 200 μm on core images in c.

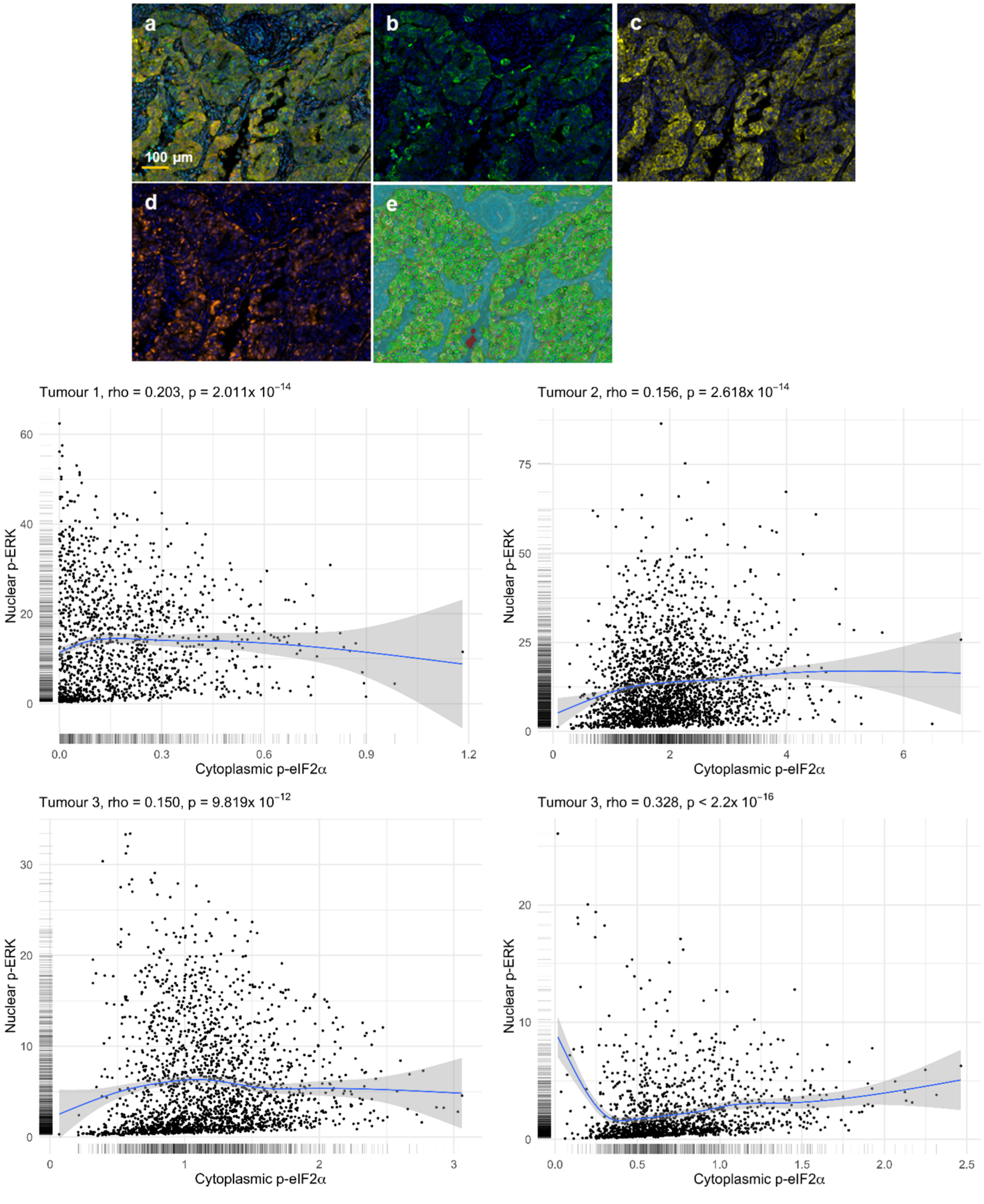

Supplementary Figure 4. **Positive correlation between cytoplasmic p-eIF2α and nuclear p-ERK in human LUAD samples.** Representative fluorescent multiplex IHC images indicating the following: (**a**) Composite image; (**b**) Cytokeratin (green) with DAPI counterstain; (**c**) p-eIF2α (yellow) with DAPI counterstain; **(d)** p-ERK with DAPI counterstain; (**e**) InForm segmentation of the tissue and subcellular compartments. Tissue is segmented into epithelium (green), stroma (blue) and background (red). Individual cells are segmented into nuclei (green) and surrounded by cytoplasm. Scatterplots show the relationship between single-cell measures of cytoplasmic p-eIF2α and nuclear p-ERK within 4 different epithelial tumor cells. Each plot also has a smoothed GAM (generalized additive model) and a measure of linear trend (Spearman's Rho and associated *P* value). Exact p values: Tumor 1 ($p = 2.011\times10^{-14}$), Tumor 2 ($p = 2.618\times10^{-14}$), Tumor 3 ($p = 9.819\times10^{-12}$) and Tumor 4 ($p < 2.2\times10^{-16}$).

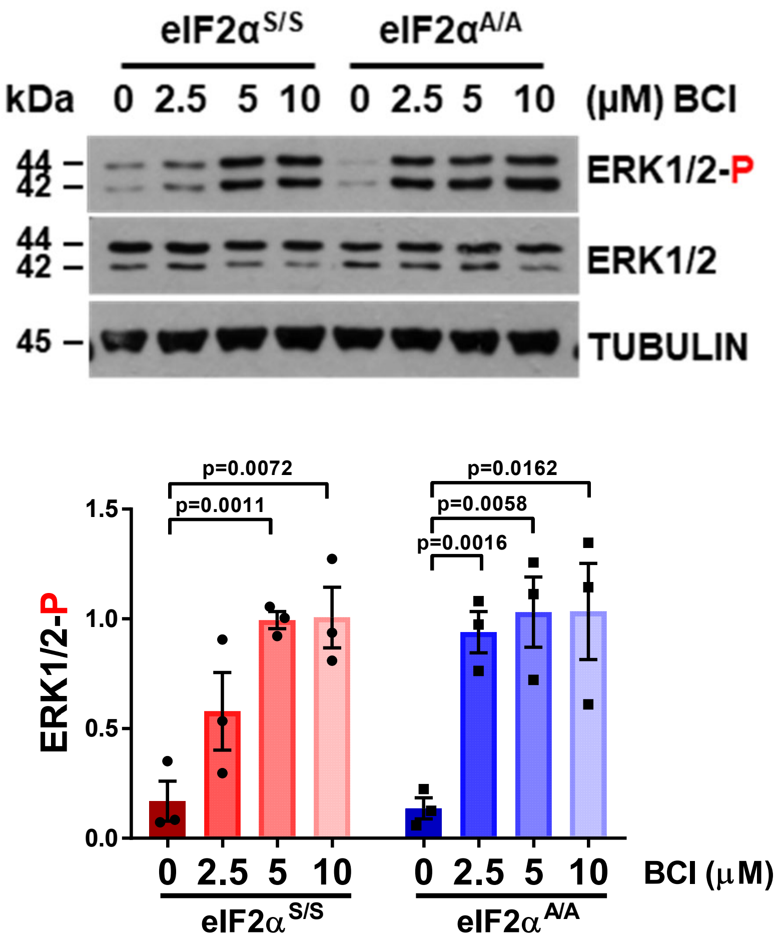

Supplementary Figure 5. The DUSP inhibitor BCI restores p-ERK levels in eIF2α$^{A/A}$ cells. Immunoblotting of mouse KRAS G12D eIF2α$^{S/S}$ and eIF2α$^{A/A}$ lung tumor cells prior to and after treatment with the indicated concentrations of BCI (μM) for 1h. Data represent mean ± SEM**.** Statistical significance was determined using two-tailed unpaired *t*-test. *P* values are indicated within the bar graphs. The quantifications of blots are from 3 biological replicates.

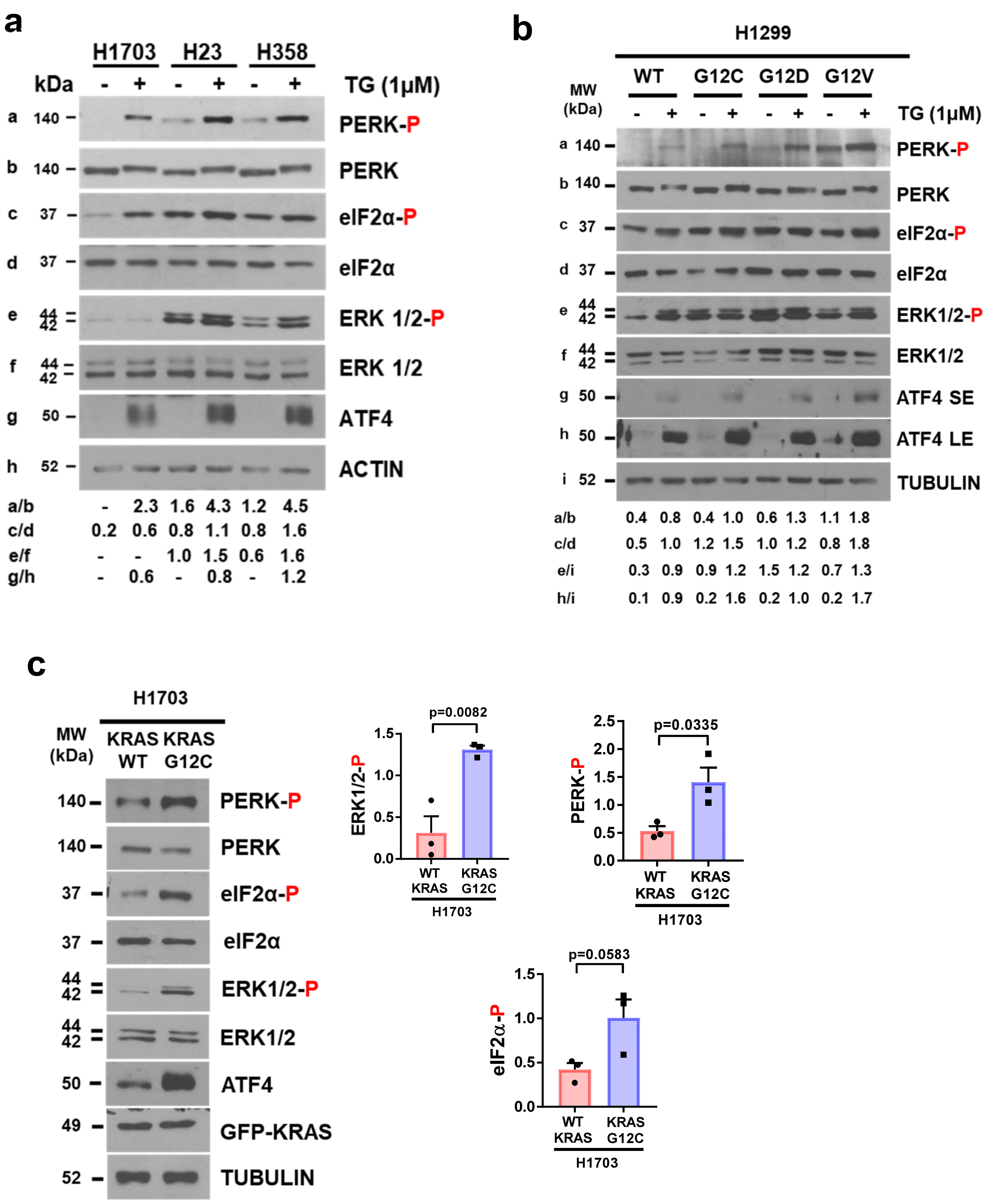

Supplementary Figure 6. Mutant KRAS upregulates the PERK/p-eIF2α arm in human LUAD cells. (**a**) Immunoblot analysis of either WT KRAS (H1703) or mutant KRAS G12C (H23, H358) LUAD cells prior to or after treatment with 1μM thapsigargin (TG) for 1.5 h. (**b**) Immunoblot analysis of H1299 cells overexpressing either WT KRAS or mutant KRAS (G12C, G12D or G12V) prior to and after treatment with 1μM TG for 1.5 h. (a-b) Blots are from 1 experiment. (**c**) Immunoblotting of H1703 cells overexpressing a GFP-tagged form of either WT KRAS or KRAS G12C. p-ERK, p-PERK and p-eIF2α were normalized to corresponding total proteins whereas DUSP6 expression was normalized to ACTIN or TUBULIN. N=3 independent experiments. Data represent mean ± SEM. Statistical significance was determined using two-tailed unpaired *t*-test. SE: Short Exposure. LE: Long Exposure.

a

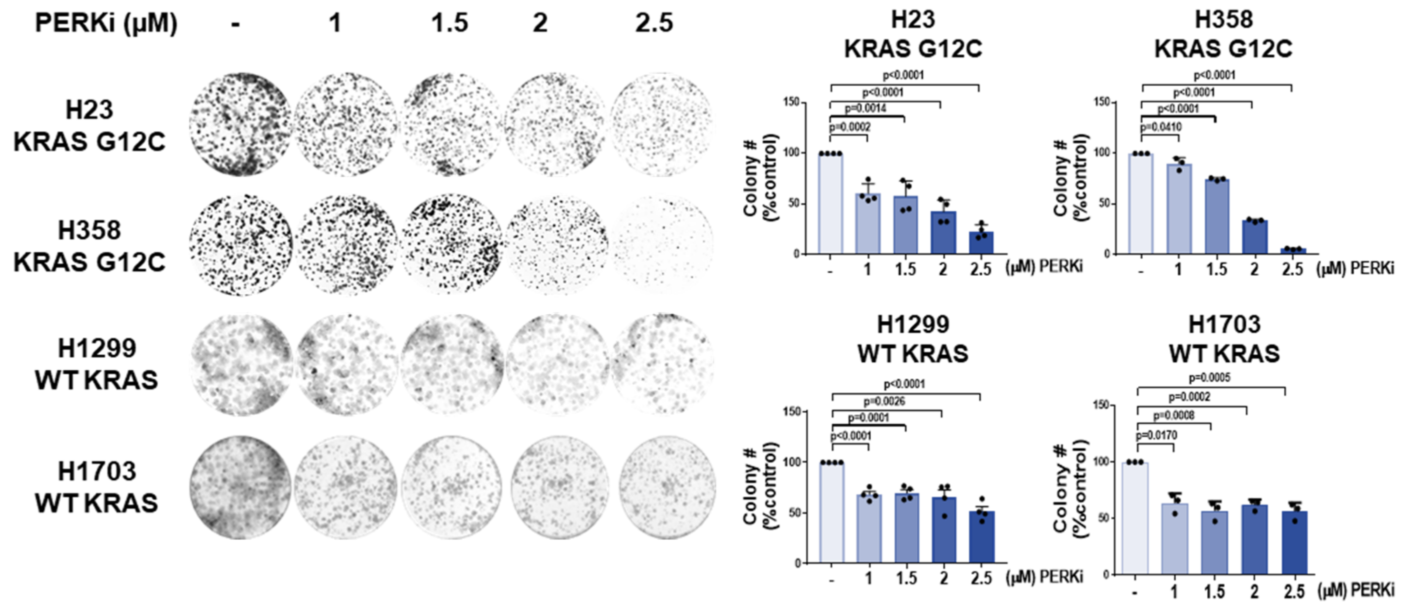

b

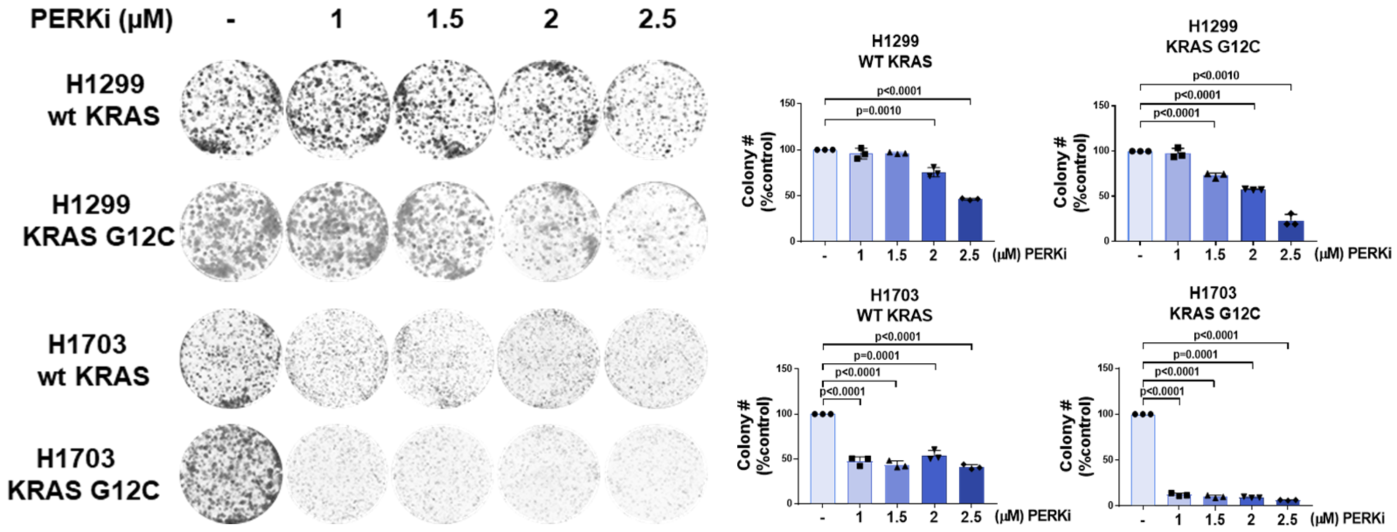

Supplementary Figure 7. Mutant KRAS sensitizes human LUAD cells to anti-proliferative effects of PERK inhibition (**a**) Colony formation assays of human LUAD cells with endogenous WT KRAS (H1299, H1703) or KRAS G12C (H358, H23) prior to and after treatment with increasing concentrations of PERK inhibitor GSK2606414 (PERKi). (**b**) Colony formation assays of H1299 and H1703 overexpressing either WT KRAS or KRAS G12C before and after treatment with increasing concentrations of PERKi. (a, b) Graphs represent data from 3 biological replicates. Data represent mean ± SEM. Statistical significance was determined using two-tailed unpaired *t*-test.

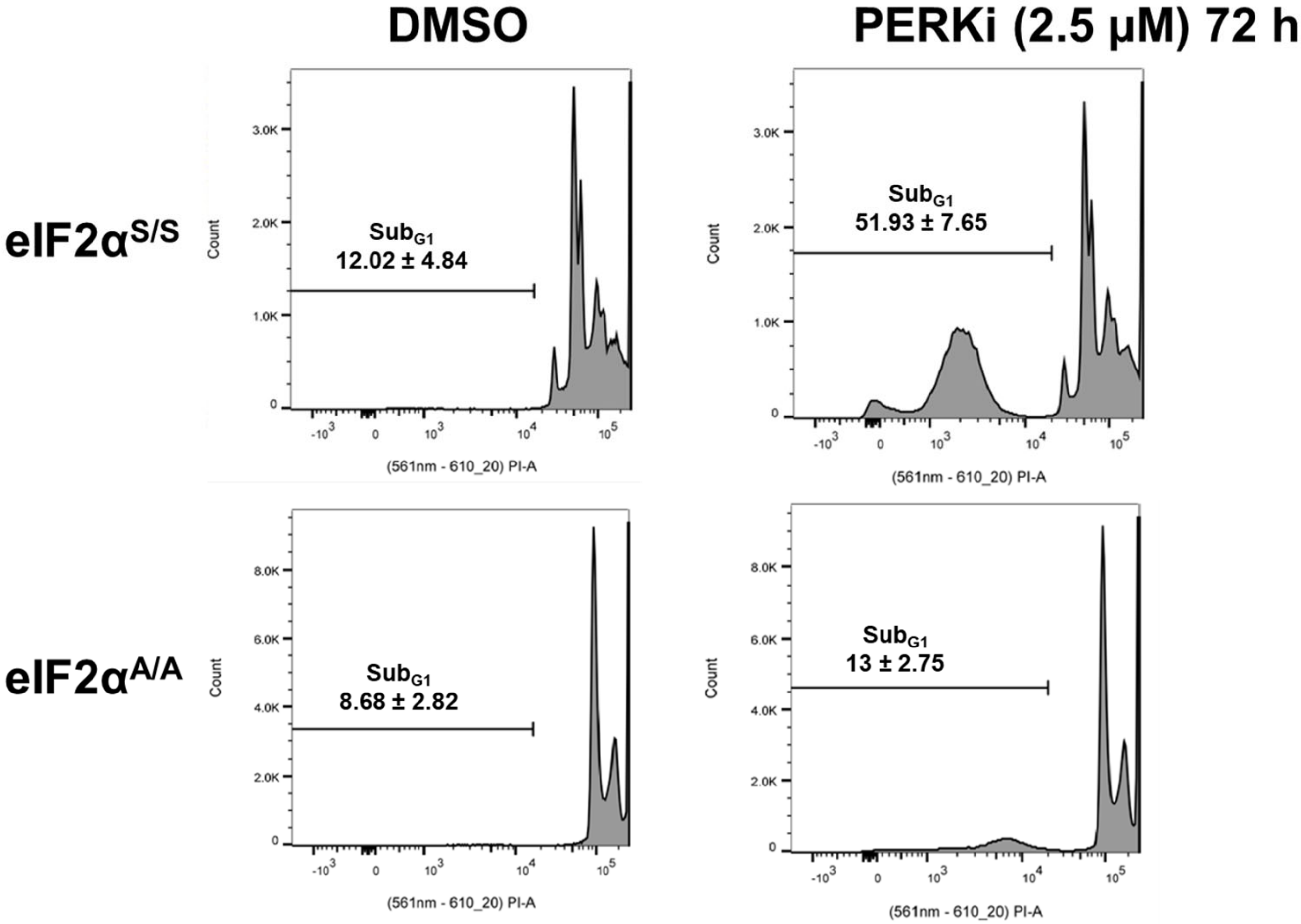

Supplementary Figure 8. PERK is pro-survival in KRAS G12D lung cancer cells. Mouse KRAS G12D eIF2α$^{S/S}$ and eIF2α$^{A/A}$ lung tumor cells were treated with PERK inhibitor GSK2606414 (PERKi) for 72 h and subjected to propidium iodide staining and flow cytometry analysis. Cell death was assessed by the analysis of cells in sub-G_1. The data represent the average of 3 biological replicates. The mean value of sub$_{G1}$ ± SEM is shown in each graph.

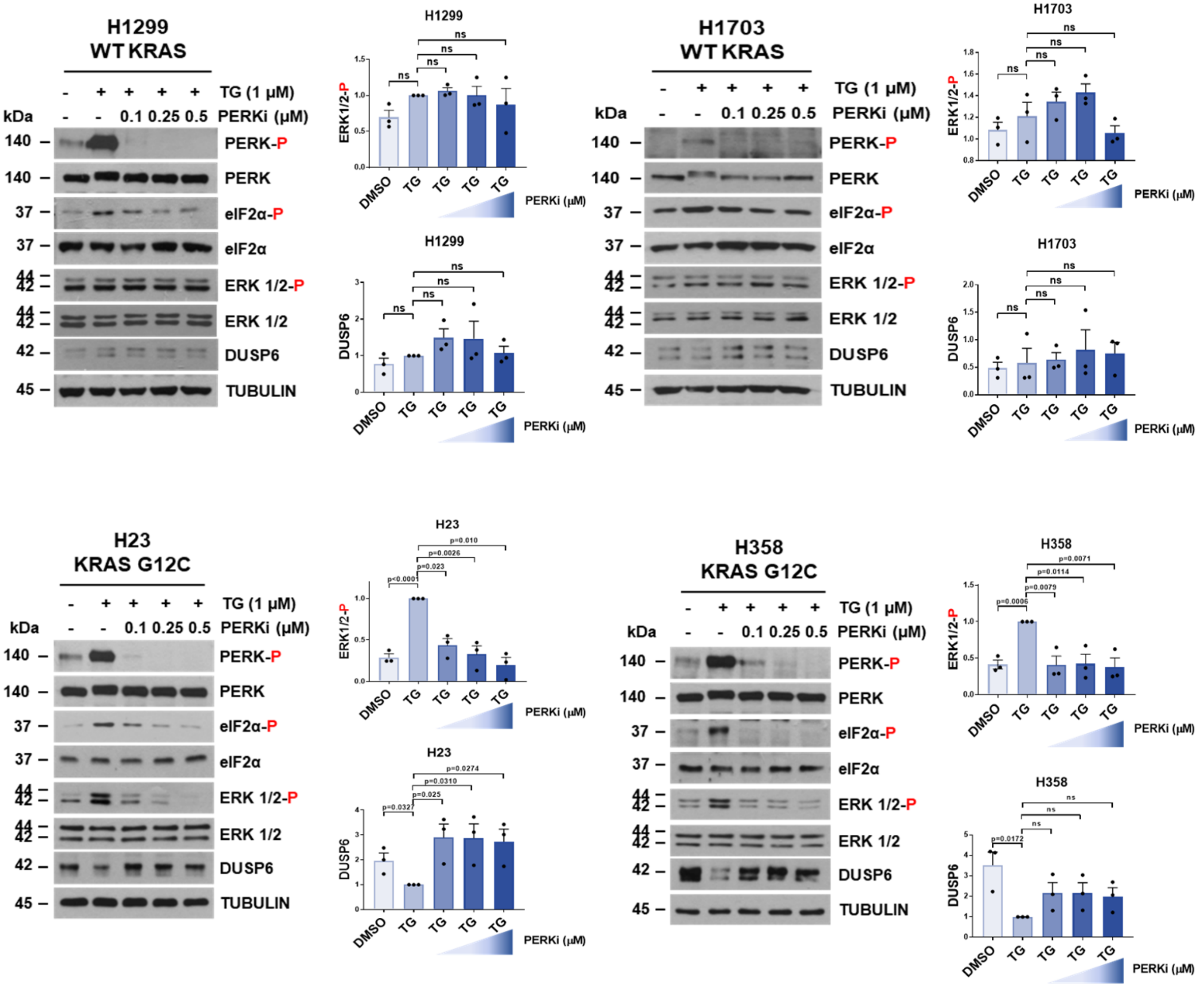

Supplementary Figure 9. The PERK/p-eIF2α arm decreases DUSP6 and stimulates p-ERK in human LUAD cells under stress. Human LUAD cells with either WT KRAS (H1299, H1703) or KRAS G12C (H23, H358) were treated with 1μM thapsigargin (TG) for 30 min followed by treatments with increasing concentration of PERK inhibitor (PERKi) GSK2606414 for 1 h. Protein extracts (50 μg) were immunoblotted for the indicated proteins. Quantification of proteins was performed from 3 biological replicates. p-ERK was normalized to total ERK whereas DUSP6 expression to ACTIN or TUBULIN. Data represent mean ± SEM. Statistical significance was determined using two-tailed unpaired *t*-test.

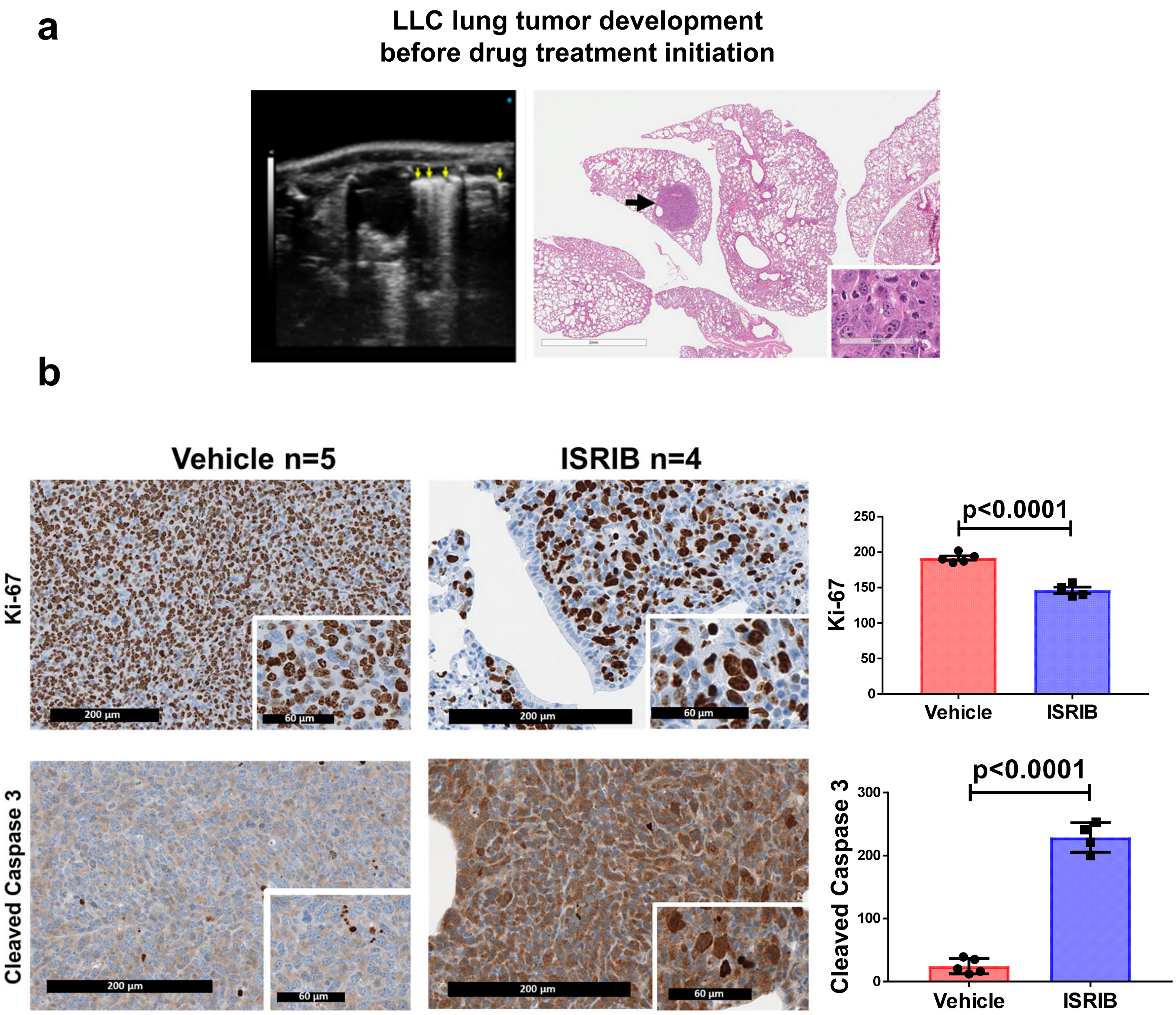

Supplementary Figure 10. ISR inhibition exhibits anti-proliferative and pro-apoptotic effects on LLC tumors in mice. (a) Representative ultrasound image (left) and H&E straining (right) indicating tumor development at day 12 after the intratracheal intubation of LLC tumors and before the initiation of ISRIB treatment. Arrow indicates the location of tumor which is magnified in the insert in the bottom right of the image. (b) IHC analysis of Ki-67 and Cleaved Caspase 3 in vehicle-control treated (n=5) and ISRIB-treated LLC tumors (n=4) in mice after 6 weeks of treatment. Data represent mean ± SEM. Statistical significance was determined using two-tailed unpaired *t*-test.

a

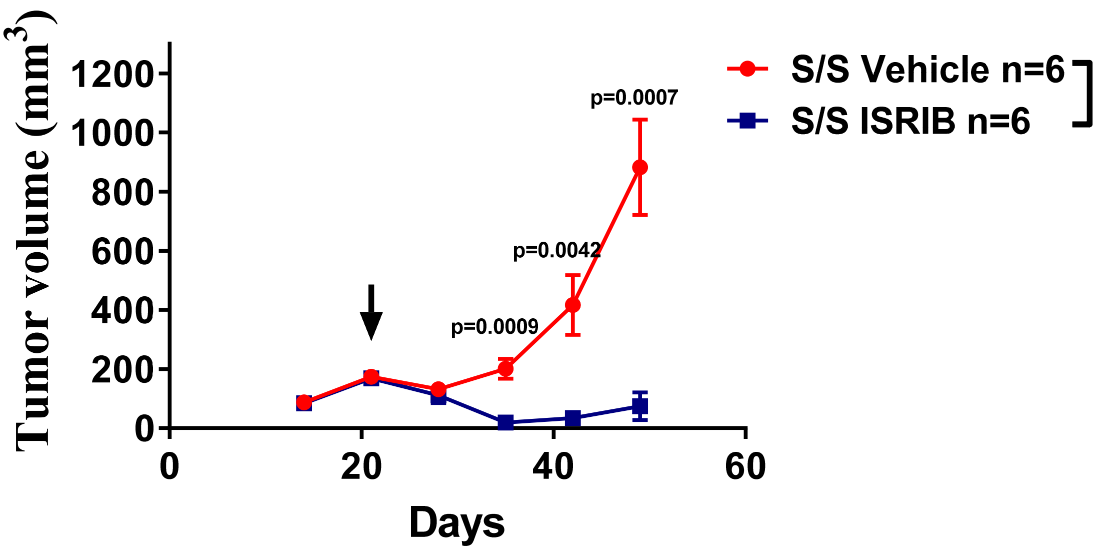

b

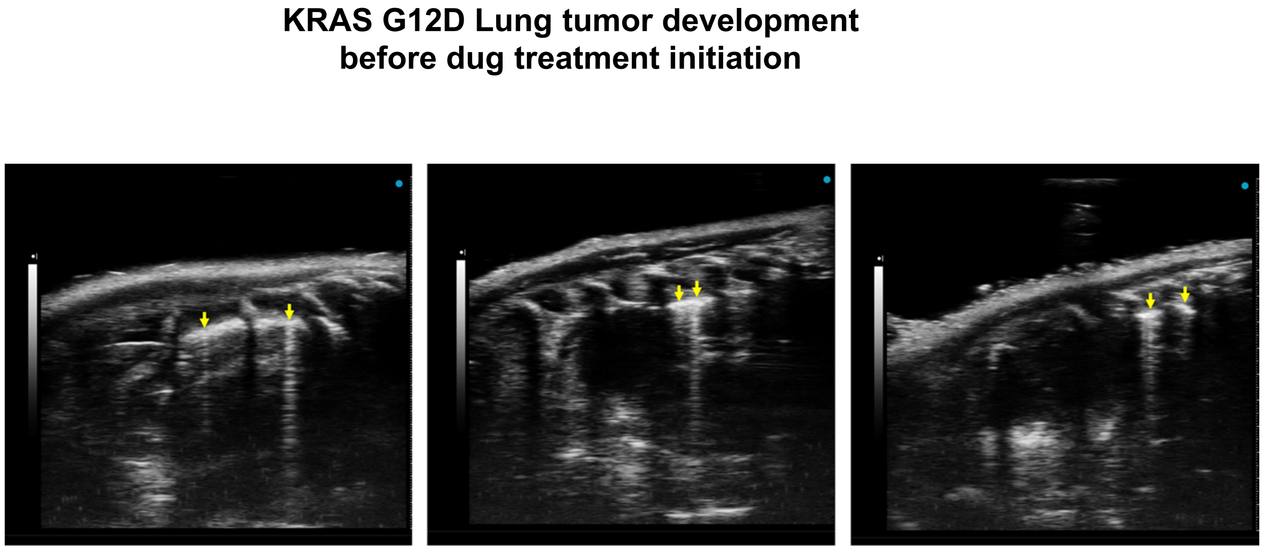

Supplementary Figure 11. Anti-tumor effects of ISRIB in mice with KRAS G12D lung tumors. (a) Mouse KRAS G12D lung cells were subjected to subcutaneous transplantation in syngeneic B6 mice. Twenty days after transplantation when sizable tumors were formed, the mice treated with either vehicle control (n=6 tumors) or 10 mg/kg of ISRIB (n=6 tumors). Arrow indicates the start of treatment (day 20). Data represent mean ± SEM Statistical significance was determined using two-tailed unpaired *t*-test. **b**) Related to Figure 6e. Representative ultrasound images of lung tumors from 3 different mice on the 10th week after induction of KRAS G12D in the mouse lungs by intratracheal intubation of CRE-expressing lentivirus and before initiation of ISRIB treatment.

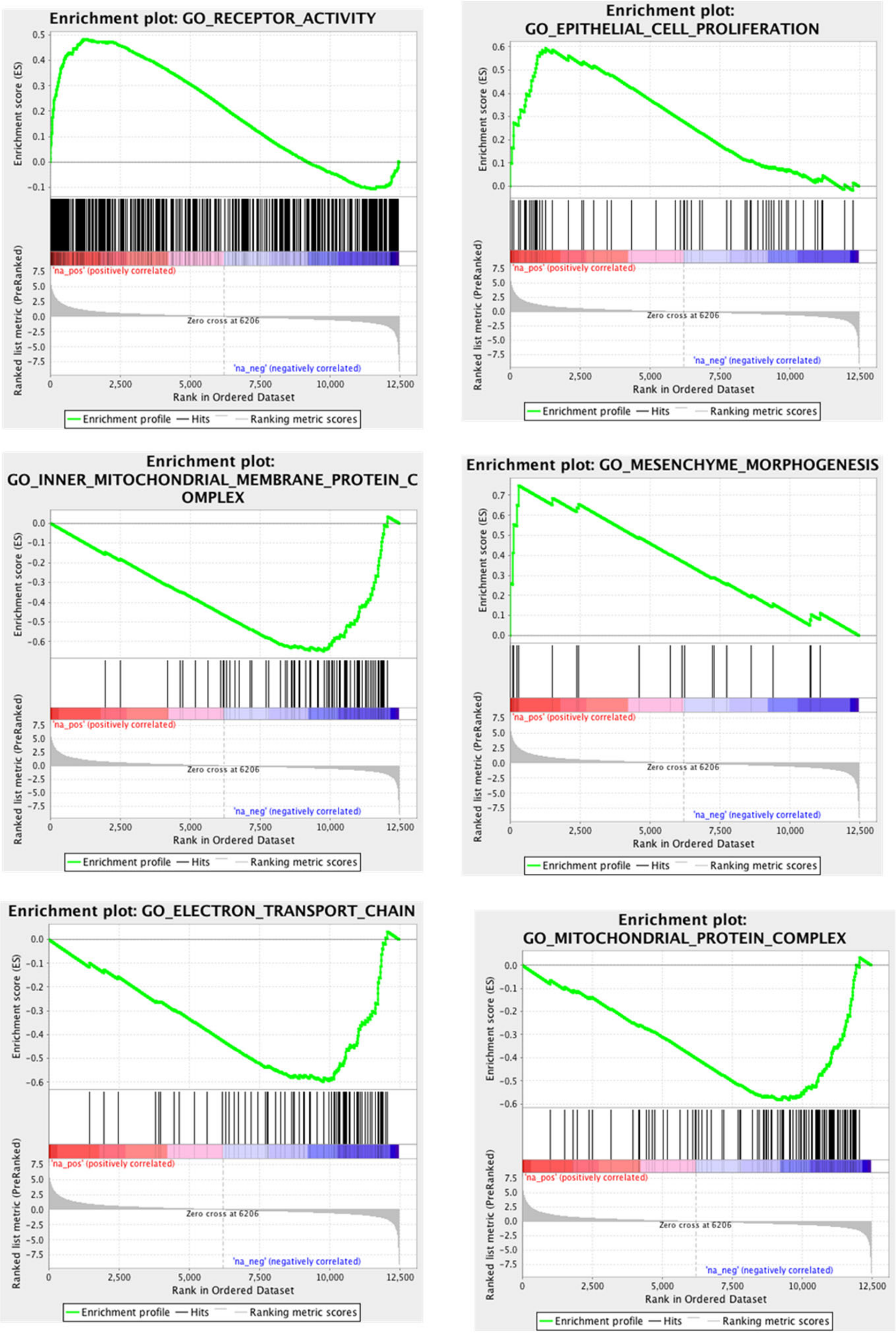

Supplementary Figure 12. p-eIF2 dictates signaling and metabolic pathways with roles in cancer formation. Gene set enrichment analyses using GO pathways in eIF2α$^{S/S}$ vs eIF2α$^{A/A}$ mouse KRAS G12D tumor cells. Show are the 6 most significantly enriched terms among up- (higher in eIF2α$^{S/S}$) or downregulated genes (higher in eIF2α$^{A/A}$).

Application	Target gene	Sequence
**RT PCR**	DUSP6	F 5'-ATA GAT ACG CTC AGA CCC GTG-3'<br>R 5'-ATC AGC AGA AGC CGT TCG TT-3'
	ACTIN	F 5'-CAGCAGATGTGGATCAGCAAG-3'<br>R 5'-GCATTTGCGGTGGACGAT-3'
	GAPDH	F 5'-GAGAGTGTTTCCTCGTCCCG-3'<br>R 5'-CAATCTCCACTTTGCCACTGC-3'
**siRNA**	eIF2AK3 (PERK)	#1 GGUUGGGUCUGAUGAAUUU<br>#2 GUUACUAUCUGCCAUACUA<br>#3 GAACGAGUCCGGAUUUUAA<br>#4 GGAAGGUCAUGGCGUUUAG
	DUSP6	#1 GAACGAUGCUUACGACAUU<br>#2 CAUCGAAUCUGCCAUUAAU<br>#3 CCUCGGACAUUGAGUCUGA<br>#4 GAAAUGGCGAUCUGCAAGA
	Non-targeting siRNA Pool	#1 UAGCGACUAAACACAUCAA<br>#2 UAAGGCUAUGAAGAGAUAC<br>#3 AUGUAUUGGCCUGUAUUAG<br>#4 AUGAACGUGAAUUGCUCAA

Supplementary Table 1. Sequence of DNA primers and siRNAs used in the study.

Antibody	Species	Company	Cat #	WB	IHC
eIF2α-P	Rabbit monoclonal	Abcam	Ab32157	1:1000	1:150
eIF2α	Mouse monoclonal	Cell signaling technology	L57A5	1:1000	
PERK-P T982	Rabbit monoclonal	Lilly Research Laboratories	PMID: 26130148	1:1000	
PERK (for human cell lines)	Rabbit monoclonal	Cell signaling technology	3192S	1:1000	
PERK (for mouse cell lines)	Mouse monoclonal	Custom made	PMID: 21954288	1:1000	
ATF4	Rabbit monoclonal	Cell signaling technology	11815S	1:1000	1:25
DUSP6	Rabbit monoclonal	Abcam	Ab76310	1:1000	1:100
ERK-P	Rabbit polyclonal	Cell signaling technology	9101S	1:1000	1:100
Actin	Mouse monoclonal	Santa Cruz Biotechnology	Sc-8432	1:1000	
α-Tubulin	Mouse monoclonal	Sigma-Aldrich, Roche	T5168	1:1000	
ERK total	Rabbit polyclonal	Cell Signaling Technology	9102S	1:1000	
Ki67	Rabbit polyclonal	Abcam	15580		1:500
Cleaved Caspase 3 Asp175	Rabbit polyclonal	Cell Signaling	9661		1:150
Mouse IgG-horseradish peroxidase-conjugated	Goat	KPL	474-1806	1:2000	
Rabbit IgG-horseradish peroxidase-conjugated	Goat	Jackson ImmunoResearch	111-035-144	1:2000	
Biotinylated anti-rabbit IgG	Horse	Vector	BA-1100		1:150

Supplementary Table 2. Antibodies for immunoblotting and IHC analyses of mouse lung tumors.

Target Protein	Primary Antibody	Secondary Antibody	Detection
Cytokeratin	Novocastra TM Liquid Mouse Monoclonal Antibody Multi-Cytokeratin<br><br>Leica, NCL-L-AE1/AE3<br><br>1:250<br><br>28 mins, 37°C	DISCOVERY OmniMap Anti-Mouse HRP<br><br>Roche, 760-4310<br><br>16 mins, 37°C	Opal 650<br><br>Akoya, FP149600 1KT<br><br>1:300<br><br>8 mins, 37°C
eIF2α-P	Phospho-eif2α (Ser51)(D9G8)XP® Rabbit mAb.<br><br>Cell Signalling Technology, 3398<br><br>1:25<br><br>6 hours, room temperature	DISCOVERY OmniMap Anti-Rabbit HRP<br><br>Roche, 760-4311<br><br>32 mins, 37°C	Opal 570<br><br>Akoya, FP148800 1KT<br><br>1:400<br><br>8 mins, 37°C
ERK-P	Phosphor-p44/42 MAPK (Erk1/2) (Thr202/Tyr204) (D13.14.4E)XP® Rabbit mAB<br><br>Cell Signalling Technology, 4370<br><br>1:100<br><br>44 mins, 37°C	DISCOVERY Roche UltraMap Anti-Rabbit HRP<br><br>Roche, 760-4315<br><br>12 mins, 37°C	Opal 520<br><br>Akoya, FP148700 1KT<br><br>1:75<br><br>8 mins, 37°C

Supplementary Table 3. Antibodies used for IHC of specimens from LUAD patients.
