# Supplementary material for: The integrated stress response is tumorigenic and constitutes a therapeutic liability in KRAS-driven lung cancer
Source: Nat Commun. 2021 Jul 30;12:4651. doi: 10.1038/s41467-021-24661-0 (PMC8324901; doi:10.1038/s41467-021-24661-0)
Supplement: Supplementary file 3 — Reporting Summary [file 41467_2021_24661_MOESM3_ESM.pdf]

nature research

Corresponding author(s): Dr. Antonis Koromilas/Dr. John LeQuesne

Last updated by author(s): Jun 1, 2021

Reporting Summary

Nature Research wishes to improve the reproducibility of the work that we publish. This form provides structure for consistency and transparency in reporting. For further information on Nature Research policies, see our Editorial Policies and the Editorial Policy Checklist.

Statistics

For all statistical analyses, confirm that the following items are present in the figure legend, table legend, main text, or Methods section.

n/a	Confirmed	
☐	☒	The exact sample size (n) for each experimental group/condition, given as a discrete number and unit of measurement
☐	☒	A statement on whether measurements were taken from distinct samples or whether the same sample was measured repeatedly
☐	☒	The statistical test(s) used AND whether they are one- or two-sided<br>*Only common tests should be described solely by name; describe more complex techniques in the Methods section.*
☐	☒	A description of all covariates tested
☐	☒	A description of any assumptions or corrections, such as tests of normality and adjustment for multiple comparisons
☐	☒	A full description of the statistical parameters including central tendency (e.g. means) or other basic estimates (e.g. regression coefficient) AND variation (e.g. standard deviation) or associated estimates of uncertainty (e.g. confidence intervals)
☐	☒	For null hypothesis testing, the test statistic (e.g. F, t, r) with confidence intervals, effect sizes, degrees of freedom and P value noted<br>*Give P values as exact values whenever suitable.*
☒	☐	For Bayesian analysis, information on the choice of priors and Markov chain Monte Carlo settings
☒	☐	For hierarchical and complex designs, identification of the appropriate level for tests and full reporting of outcomes
☐	☒	Estimates of effect sizes (e.g. Cohen's d, Pearson's r), indicating how they were calculated

Our web collection on statistics for biologists contains articles on many of the points above.

Software and code

Policy information about availability of computer code

Data collection	VisualSonics VEVO 3100 high frequency ultrasound for detection of lung tumors in mice<br>ISCO model 160 Gradient Former and density gradient fractionation system<br>Aperio Scanscope AT2 Turbo scanner of tumor sections (Leica Biosystems)<br>Roche DISCOVERY Ventana® platform for IHC analysis<br>Hamamatsu NanoZoomer-XR C12000 tumor slide scanner<br>Akoya Vectra® tumor slide scanner<br>Visiopharm® digital pathology platform<br>BD LSRFortessa Flow Cytometer
Data analysis	ImageJ 1.51e for quantification of immunoblottings<br>anota2seq package (1.8.0) for analysis of gene expression data<br>Ingenuity Pathway Analysis (IPA)/QIAGEN digital insights for analysis of gene expression data<br>Gene set enrichment analysis (GSEA v4.0.3, Broad Institute) for analysis of gene expression data<br>Gene Ontology geneset v5.2 (MSigDB) for analysis of gene expression data<br>Aperio Imagescope software version 12.3.3 (Leica Biosystems) for analysis of histology mages<br>Akoya inform Advanced Image Analysis software (analysis of tumor regions)<br>RStudio (1.0.153) software for statistical analysis of tumor imaging data<br>FACSDiva abd FlowJo software for analysis of cells by Flow Cytometer

For manuscripts utilizing custom algorithms or software that are central to the research but not yet described in published literature, software must be made available to editors and reviewers. We strongly encourage code deposition in a community repository (e.g. GitHub). See the Nature Research guidelines for submitting code & software for further information.

Data

Policy information about availability of data

All manuscripts must include a data availability statement. This statement should provide the following information, where applicable:

- Accession codes, unique identifiers, or web links for publicly available datasets
- A list of figures that have associated raw data
- A description of any restrictions on data availability

The RNA seq data generated in this study have been deposited in the Gene Expression Omnibus (GEO) GSE155238; GEO Accession viewer (nih.gov)). The remaining data are available within the Article, Supplementary Information or available from the authors upon request.

Field-specific reporting

Please select the one below that is the best fit for your research. If you are not sure, read the appropriate sections before making your selection.

☒ Life sciences ☐ Behavioural & social sciences ☐ Ecological, evolutionary & environmental sciences

For a reference copy of the document with all sections, see nature.com/documents/nr-reporting-summary-flat.pdf

Life sciences study design

All studies must disclose on these points even when the disclosure is negative.

Sample size	Sample size for mouse experiments was based on previous studies with the same KRAS/TP53 mouse model of lung cancer where the use at least 5 mice per group to generate statistical significant data (e.g. PMID: 15093544; PMID: 30867319; PMID: 24618618). The number of mice included in our studies yielded statistically significant differences in tumor progression and treatment with ISR inhibitors as indicated in the article. Analysis of tumors in mouse lungs by Ultrasound imaging was performed with at least 3 mice per mouse type per treatment yielding statistically significant results. For in vitro experiments with tumor sections or cells in culture, we used different time points and conditions of treatments to obtain statistically significant responses to anti-tumor drugs and regulation of ERK phosphorylation and DUSP6 expression.
Data exclusions	There were no exclusions of data.
Replication	We performed experiments with independent samples and all attempts at replication were successful. We included several biological replicates as indicated in the figures to ensure reproducibility and statistical significance of the data.
Randomization	In the animal studies mice from each breeding cohort were selected in the experimental groups in a random fashion. The pathology and IHC analyses of tumor sections from each experimental group was performed randomly.
Blinding	The treatments of mice with ISR inhibitors could not be blinded because the whole process required the knowledge of experimental groups subjected to treatments. However, analysis of tumor progression by Ultrasound imaging was blinded and performed by two lab personnel independently (H. Ghaddar, S. Wang). Also, a IHC of lung tumors was performed by technicians at the Pathology Unit of our Core facility, who were unaware of the nature of our experiments and identity of the samples.

Reporting for specific materials, systems and methods

We require information from authors about some types of materials, experimental systems and methods used in many studies. Here, indicate whether each material, system or method listed is relevant to your study. If you are not sure if a list item applies to your research, read the appropriate section before selecting a response.

Materials & experimental systems

n/a	Involved in the study	
☐	☒	Antibodies
☐	☒	Eukaryotic cell lines
☒	☐	Palaeontology and archaeology
☐	☒	Animals and other organisms
☐	☒	Human research participants
☒	☐	Clinical data
☒	☐	Dual use research of concern

Methods

n/a	Involved in the study	
☒	☐	ChIP-seq
☐	☒	Flow cytometry
☒	☐	MRI-based neuroimaging

Antibodies

Antibodies used	eIF2α-P rabbit monoclonal Abcam Ab 32157<br>eIF2α mouse monoclonal Cell Signaling Technology L57A5<br>phospho-PERK T982 rabbit monoclonal Lilly Research Laboratories (PMID: 26130148)

PERK rabbit monoclonal Cell Signaling Technology 3192S
PERK mouse monoclonal PMID: 21954288
ATF4 rabbit monoclonal Cell signaling Technology 11815S
DUSP6 rabbit polyclonal Cell signaling technology ab76310
phospho-ERK rabbit polyclonal Abcam Ab76310
Actin mouse monoclonal Santa Cruz Biotech. SC-8432
alpha-Tubulin mouse monoclonal Sigma-Aldrich Roch T5168
ERK rabbit polyclonal Cell Signaling Technology 9102S
Ki67 rabbit polyclonal Abcam Ab15580
Cleaved Capase 3 (Asp175) rabbit polyclonal Cell signaling 9661
Mouse IgG-HRP goat monoclonal KPL 474-1806
rabbit IgG-HRP goat monoclonal Jackson ImmunoResearch 111-035-144
Biotinylated anti-rabbit IgG horse monoclonal Vector BA-1100

Validation

The antibodies were validated in prior studies and passed the quality control assays of the manufacturers as indicated in the websites of the providing companies.

Eukaryotic cell lines

Policy information about cell lines

Cell line source(s)

Primary KRAS G12D eIF2αS/S and eIF2αA/A lung tumor cells were isolated from mice at 20 weeks of lung tumor formation. Cells were frozen and different stocks were used in experiments. H1299 cells overexpressing either WT KRAS 4B or mutant KRAS 4B proteins were provided by Dr. M. Broggini (co-author of the study). H1703, H1299 and H23 were obtained from Dr. S. Huang (Biochemistry, McGill University) and H358 cell lines were obtained from Dr. M. Witcher (Biochemistry, McGill University). LLC cells were obtained from Dr. S. Wing (Endocrinology, McGill University).

Authentication

Cells were authenticated in previous publications. Cell lines with KRAS G12C were authenticated by treatments with AMG510 inhibitor followed by detection of inhibition of MAPK signaling.

Mycoplasma contamination

Cells were mycoplasma negative and maintained in mycoplasma free conditions.

Commonly misidentified lines
(See ICLAC register)

No misidentified lines were used.

Animals and other organisms

Policy information about studies involving animals; ARRIVE guidelines recommended for reporting animal research

Laboratory animals

We used mice in C57BL/6 background in all experiments. For lung tumor induction, we used randomly selected male and female mice 2 months old. Mice were maintained in ventilated cages under 40-60% humidity with a 12 hour dark light cycle.

Wild animals

Wild animals were not included in the study.

Field-collected samples

Field-collected samples were not used in the study.

Ethics oversight

All experiments with mice were performed according to Animal Welfare Committee of McGill University (protocol #5754)
The usage of human samples was approved by Northampton Research Ethics Committee (reference 14/EM/1159) and University Hospitals Leicester NHS Trust Research and Innovation Department (reference UHL 11363).

Note that full information on the approval of the study protocol must also be provided in the manuscript.

Human research participants

Policy information about studies involving human research participants

Population characteristics

Describe the covariate-relevant population characteristics of the human research participants (e.g. age, gender, genotypic information, past and current diagnosis and treatment categories). If you filled out the behavioural & social sciences study design questions and have nothing to add here, write "See above."

Recruitment

Describe how participants were recruited. Outline any potential self-selection bias or other biases that may be present and how these are likely to impact results.

Ethics oversight

Identify the organization(s) that approved the study protocol.

Note that full information on the approval of the study protocol must also be provided in the manuscript.

Flow Cytometry

Plots

Confirm that:

☐ The axis labels state the marker and fluorochrome used (e.g. CD4-FITC).

☒ The axis scales are clearly visible. Include numbers along axes only for bottom left plot of group (a 'group' is an analysis of identical markers).

☐ All plots are contour plots with outliers or pseudocolor plots.

☒ A numerical value for number of cells or percentage (with statistics) is provided.

Methodology

Sample preparation	Samples were KRAS G12D eIF2αS/S and eIF2αA/A cells isolated from mice and maintained in culture. The cells were treated with the PERK inhibitor GSK2606414, fixed and stained with propidium iodide (PI)and analyzed by FACS analysis by the identification of the population of dead cells in the sub-G1 phase.
Instrument	FACS data was collected using BD LSRFortessa flow cytometer.
Software	FACS data was collected using FACSDiva and analyzed using FlowJo.
Cell population abundance	FACS analysis was performed on each sample with a total cell number of > 50,000 events.
Gating strategy	All samples were gated for FSC/SSC. Gates were drawn from distinct, observable stained populations. Gating was done for below G1.

☒ Tick this box to confirm that a figure exemplifying the gating strategy is provided in the Supplementary Information.
